# Differential diagnosis of COVID-19 in symptomatic patients at the University Hospital Center Mohammed VI, Marrakesh

**DOI:** 10.11604/pamj.2020.36.269.24558

**Published:** 2020-08-12

**Authors:** Khadija Krati, Jihane Rizkou, Adil Ait Errami, Lamiaa Essaadouni

**Affiliations:** 1 Department of Gastroenterology, University Hospital Center Mohammed VI, Marrakech, Morocco,; 2 Department of Internal Medicine, University Hospital Center Mohammed VI, Marrakech, Morocco

**Keywords:** COVID-19, respiratory infections, respiratory tract diseases, differential diagnosis

## Abstract

**Introduction:**

coronavirus disease 2019 caused by severe acute respiratory syndrome coronavirus 2 was first reported in Wuhan, China. Clinical spectrum of this disease has nonspecific symptoms shared by many other frequent infectious diseases of the respiratory tract and other respiratory tract diseases. This study explains the importance of differential diagnosis between COVID-19 and other lung diseases.

**Methods:**

we analyzed in this study, the demographic features, clinical presentations, laboratory data and radiologic findings of the COVID-19 patients in comparison to those with other respiratory infections or diseases.

**Results:**

the mean age of all patients was 38.04 years; 35 patients were later confirmed to be positive for SARS-CoV-2 infection. The most common symptoms reported by both groups included nonproductive cough and myalgia. Two of the non-COVID-19 patients were having below 92% oxygen saturation and low systolic blood pressure. The patients shared relatively similar laboratory findings except 3% of the non-COVID-19 patients who had lympho-neutropenia and 22.6% had high levels of C-reactive protein. Pulmonary tuberculosis and autoimmune disease respiratory disorder were suspected in 2 of the non-COVID-19 patients respectively.

**Conclusion:**

we emphasize the importance of good screening protocols, rapid detection of SARS-CoV-2 and other most common respiratory pathogens, which may help for a better control of COVID-19 spread and avoid delayed care of other lung diseases.

## Introduction

Coronavirus disease 2019 (COVID-19) is caused by severe acute respiratory syndrome coronavirus 2 (SARS-CoV-2) [1]. Its manifestations can be viewed as a combination of the 2 processes, namely viral pneumonia and acute respiratory distress syndrome (ARDS) [2]. COVID-19 is a novel disease recognized initially in Wuhan, Hubei Province, China, in December 2019 and is now pandemic. It is likely caused by zoonotic spillover of a beta-coronavirus type 2B that is now transmitted between humans. Along with the other serious coronavirus infections of SARS and Middle East respiratory syndrome (MERS), which also cause ARDS, COVID-19 represents an ongoing global threat as this virus family has the potential to mutate and infect non immune populations [2, 3]. By way of definition, a symptomatic COVID-19 case is a person who has developed signs and symptoms suggestive of COVID-19 [4]. Clinical spectrum of this disease varied from mild to severe. Multiple body tracts may be involved, including the respiratory, gastrointestinal, musculoskeletal and neurologic tracts.

However, more common symptoms are fever (83-98%), cough (76-82%), shortness of breath (31-55%) and fatigue (34%) [1]. These nonspecific symptoms are shared by many other frequent infectious diseases of the respiratory tract caused by bacteria and viruses, most of which are self-limiting but may also progress to severe conditions. Among these, the most relevant is influenza A/B, usually characterized by fever, myalgia, headache and non-productive cough, which may also cause complications with high morbidity and mortality rate, such as pneumonia, myocarditis, central nervous system disease and death [1, 5]. The purpose of our study is to show that, since the symptoms are similar to other respiratory infections, differential diagnosis of COVID-19, must include other more common infections and other respiratory tract diseases, in order not to delay their appropriate care.

## Methods

During April 3^rd^ to May 25^th^ 2020, a total of 97 patients who were admitted to 2 isolation COVID departments (gastroenterology and internal medicine) at the University Hospital Centre Mohammed VI Marrakesh; met the screening criteria and the diagnostic algorithm of COVID-19 reported by Moroccan CDC guidelines, in collaboration with Moroccan Society of Emergency Medicine and Moroccan Society of Anesthesia, Analgesia and critical care. In the isolation unit of the University Hospital emergency room, epidemic context, travel history data, clinical features of the patients were collected and naso-oropharyngeal sampling for viral identification were also completed. For cases reporting of COVID-19 infection, blood tests were performed after their admission to the isolation COVID departments. The loads of SARS-CoV-2 in the naso-oropharyngeal specimens were performed, using polymerase chain reaction (PCR) technique, upon and 24h after admission to identify the COVID-19-infected patients. However, broad screening for other common respiratory pathogens was not performed. Patients were hospitalized in the isolation COVID-19 departments and pending the results of the PCRs, symptomatic treatment was administered with close clinical monitoring. We analyzed the demographic features, clinical presentations, laboratory data, radiologic findings and travel and exposure contact histories, of the COVID-19 patients in comparison to those with other respiratory infections or diseases.

## Results

Among the patients suspected with COVID-19 in Marrakech’s Mohammed VI University Hospital Center, a total of 97 patients were admitted to 2 isolation COVID-19 departments (gastroenterology and internal medicine), 35 of them were later confirmed to be positive for SARS-CoV-2 infection. As shown in Table 1, the ages of patients ranged from 17 to 85 years (mean age of 38.04 years). There were 40 women and 57 men with a sex ratio of 1.4. None of patients were traveling to a high risk area. About 12 non-COVID-19 patients and 30 COVID-19 patients confirmed in this study had a contact history with a confirmed COVID-19 patient. Fifty three percent of the non-COVID-19 patients and 31 of the COVID-19 patients had at least one comorbidity +/- risk factor. The average length of hospital stay for non-COVID-19 patients, pending PCR results, was 8.5 days.

**Table 1:** epidemic context, travel and contact history, and symptoms of the patients suspected with COVID-19 in this study

	COVID-19 (n=35)	Non-COVID-19 (n=62)	All Patients (n=97)
**Demographic**			
Age (range), year	34.3(19-63)	40.1(17-85)	38.04(17-85)
Male	20(57%)	37(60%)	57(59%)
Female	15(43%)	25(40%)	40(41%)
**Travel/Contact history**			
Contact with confirmed COVID-19 patient	30(85%)	10(19%)	42(43%)
Contact with a person who has stayed in a high risk zone	2(5.7%)	2(3.2%)	4(4.1%)
Travel to a high risk zone	0	0	0
**Co-morbidities and risk factor**			
Age >70years	0	7(7.2%)	7(7.2%)
Diabetes	4(4.1%)	10(10.3%)	14(14.4%)
High blood pressure	1(1%)	6(6.2%)	7(7.2%)
Coronary artery disease	0	0	0
Heart disease	0	2(2%)	2(2%)
Chronic Airway disease	0	0	0
Tuberculosis	0	1(1%)	1(1%)
Liver cirrhosis	1(1%)	0	1(1%)
Autoimmune disease	3(3%)	6(6.2%)	9(9.2%)
Malignancy	0	4(4.1%)	4(4.1%)
Renal failure	0	2(2%)	2(2%)
Immunosuppression	0	1(1%)	1(1%)
BMI >40	0	0	0
Smoking +/- alcohol intake	5(5.1%)	15(15.4%)	20(20.6%)
**Symptoms**			
Fever ≥38**°**C	2(2%)	13(13.4%)	15(15.4%)
Nonproductive cough	20(20.6%)	30(31%)	50(51.5%)
Productive cough	2(2%)	5(5.1%)	7(7.2%)
Asthenia	10(10.3%)	35(36%)	45(46.3%)
Dyspnea	0	5(5.1%)	5(5.1%)
Chest tightness	1(1%)	10(10.3%)	11(11.3%)
Chest pain	0	3(3%)	3(3%)
Stuff nose	0	3(3%)	3(3%)
Sore throat	0	0	0
Rhinorrhea	2(2%)	2(2%)	4(4.1%)
Headache	4(4.1%)	15(15.4%)	19(19.5%)
Myalgia	10(10.3%)	40(41.2%)	50(51.5%)
Abdominal pain	3(3%)	5(5.1%)	8(8.2%)
Diarrhea	3(3%)	7(7.2%)	10(10.3%)
Nausea/Vomiting	4(4.1%)	0	4(4.1%)
Anosmia	2(2%)	1(1%)	3(3%)
Dysgeusia	2(2%)	1(1%)	3(3%)

**Clinical features, laboratory and imaging data of COVID-19 patients in comparison with non-COVID-19 group:** the most common symptoms reported by both groups included nonproductive cough and myalgia (51.5%), followed by asthenia (46.3%), headache (19.5%), fever (15.4%) and chest tightness (11.3%) (Table 1). For clinical features, as shown in Table 2, 3.2% (n=2) of the non-COVID-19 patients were having below 92% oxygen saturation and low systolic blood pressure. 6.5% (n=4) were polypneic and 9.6% (n = 6) were having tachycardia. On the other hand, COVID-19 patients had relatively stable clinical features with only 5.7% (n = 2) of theme having a fever. To note that none of the patients in this study were having impaired consciousness. Chest computed tomography (CT) scan was performed in 28 of the COVID-19 patients, showing characteristic abnormalities in 100% (ground-glass opacity typically multifocal, bilateral, asymmetrical and predominates in peripheral, posterior and basal regions). In the non-COVID-19 patients, chest CT was performed in 32% (n=20). In 10 patients, CT scan as normal, interstitial infiltrates was noticed in 2 patients, unilateral subpleural atypical ground-glass opacity in 4 patients, alveolar syndrome in 2 patients, aspects of TBK complicated with bilateral pneumothorax in 1 patient and aspect of emphysema and bronchiectasis in 1 patient. In the COVID-19 patients, 85.7% had normal white blood cell (WBC) counts, while 14.3% had leukocytosis without lymphocytopenia (Table 3). Of the non-COVID-19 patients, 77.4% had normal WBC counts and 3% had lympho-neutropenia, while 19.3% had leukocytosis and 22.6% had high levels of C-reactive protein (CRP).

**Table 2:** clinical features of the patients suspected with COVID-19 in this study

Clinical features	COVID-19 (n=35)	Non-COVID-19 (n=62)
**Heartbeat (ppm**)		
>100 (tachycardia)	0	6(9.6%)
60-100	35(100%)	56(90.4%)
<60 (bradycradia)	0	0
**Respiratory rate (cpm)**		
<20	35(100%)	58(93.5%)
>25	0	4(6.5%)
**Oxygen saturation%**		
>95	35(100%)	60(96.8%)
92-94	0	0
<92	0	2(3.2%)
**Temperature**		
>38**°**C (fever)	2(5.7%)	13(21%)
<38**°**C	33(94.3%)	49(79%)
**Blood pressure mm-Hg (systolic)**		
Normal	35(100%)	60(96.8%)
<90	0	2(3.2%)
**State of consciousness**		
Normal	35(100%)	62(100%)
Impaired consciousness	0	0

**Table 3:** laboratory data of the patients suspected with COVID-19 in this study

	COVID-19 (n=35)	Non-COVID-19 (n=62)
**WBC, 103/uL (3600 -11000)**		
<3600	0	2(3.3%)
3600-11000	30(85.7%)	48(77.4%)
>11000	5(14.3%)	12(19.3%)
**Neutrophil, 103/uL**		
<2000 (neutropenia)	0	2(3.3%)
2000 -7000	30(85.7%)	48(77.4%)
>7000	5(14.3%)	12(19.3%)
**Lymphocytes, 103/uL**		
<1000 (lymphopenia)	0	2(3.3%)
>1000	35(100%)	60(96.7%)
**Creatinine, mg/L (7-12)**		
Normal	34(97%)	57(92%)
>12	1(3%)	5(8%)
**Aspartate aminotransferase, IU/L (13-39)**		
Alanine aminotransferase, IU/L (5-40)	29(18-40)	38(15-60)
C-reactive protein, mg/L (<12)	23(7-42)	28(6-50)
<12	30(85.7%)	48(77.4%)
>12	5(14.3%)	14(22.6%)
Lactate dehydrogenase, U/L (140-271)	215(170-260)	224(147-300)
**PT, % (70-100)**		
<70	0	2(3.3%)
Normal	35(100%)	60(96.7%)

Six patients total had high levels of creatinine, no statistically significant differences were found between the COVID-19 and non-COVID-19 groups in levels aspartate aminotransferase (AST), alanine aminotransferase (ALT) and lactate dehydrogenase (LDH). For The COVID-19 patients, the treatment was followed and completed according to the established common protocol by the Moroccan CDC guidelines (chloroquine or hydroxy-chloroquine sulfate in combination with azithromycin). All the COVID-19 patients completed the protocol, were declared cured, after 2 control PCRs and were confined to a hotel, for an additional 14 days. For the non-COVID-19 patients, 51 of them were discharged from the COVID-19 departments after a negative PCR +/- normal chest CT scan, highly suspecting a common non-COVID-19 respiratory infection. For these patients, their health statuses were very stable and have had symptomatic treatment only. For the rest of the non-COVID-19 patients: pulmonary tuberculosis was suspected in 2 patients. One patient had a congestive colitis, one patient had bronchiectasis, one patient had spinal cord aplasia, 2 patients were suspected of having autoimmune disease respiratory disorder, 1 patient had palliative stage pulmonary tumor, 1 patient on pulmonary tuberculosis treatment was complicated by a bilateral pneumothorax and has been transferred to the intensive care unit (ICU) for further care and 1 patient had acute lung edema and has been transferred to a hemodialysis unit, while 2 patients died including a patient with a vaterian ampuloma.

## Discussion

COVID-19 is a severe and easily transmissible infectious disease spreading all around the world. Understanding the clinical symptoms of COVID-19 is important; the clinical symptoms are indicated nonspecific. Common symptoms include fever, cough and myalgia or fatigue. Patients may initially present with diarrhea and nausea a few days prior to fever, suggesting fever is dominant but not the premier symptom of infection. A small number of patients can have headache or hemoptysis [6, 7] and even relatively be asymptomatic [8, 9]. Therefore, the contact and travel history were the main screening criteria for SARS-CoV-2 in the early phase of COVID-19 illness, as no specific symptoms or laboratory data were noted in comparison with non-COVID-19 patients [1]. Clinical manifestations of viral respiratory infections range from symptomatic or flu-like symptoms such as fever, nonproductive or productive cough, myalgia, rhinorrhea and sore throat, to acute respiratory distress syndrome (ARDS) or multiple organs failure [1, 10]. Nonspecific symptoms are shared by many other frequent infectious and non-infectious diseases of the respiratory tract. Most common pathogens were influenza A/B, adenovirus and respiratory syncytial virus, without forgetting bacterial pathogens (streptococcus pneumonia, mycoplasma and chlamydia, tuberculosis...), bronchopulmonary tumors, respiratory lesion of systemic diseases and other diseases of the respiratory tract. Thus, the lack of specificity for clinical symptoms, laboratory data, makes differential diagnosis of this disease from other viral respiratory infections difficult [1].

Radiological examinations are of great importance in the early detection and management of COVID-19. Because chest radiography is not sensitive for the detection of ground-glass opacity (GGO) and may demonstrate normal findings in early stage of infection, it was no recommended as the first-line imaging modality for COVID-19. Therefore, thin-slice chest CT plays a vital role in early detection; observation and disease evaluation. Coronavirus disease 2019 has different imaging manifestations at different stages, which are mainly related to pathogenesis, typical CT findings of COVID-19 include peripherally distributed multifocal ground-glass opacities (GGOs) with patchy consolidations and posterior part or lower lobe involvement predilection [11]. In imaging diagnosis, COVID-19 is difficult to distinguish from pneumonias caused by influenza A virus, influenza B virus, cytomegalovirus, adenovirus, respiratory syncytial virus, SARS-CoV, MERS coronavirus and other viral pneumonias as well as bacterial pneumonia. Viral pneumonias other than COVID-19 mainly manifest as peribronchial and perivascular interstitial inflammation that moves toward the inner part of the pulmonary interstitium. Coronavirus disease 2019 also needs to be differentiated from bacterial pneumonia, mycoplasma pneumonia (MPP) and chlamydia pneumonia 9. Bacterial pneumonia occurs in the lung parenchyma and mainly manifests as bronchial pneumonia or lobar pneumonia with many inflammatory secretions in the bronchioles and alveoli [12].

Although various types of pneumonias have certain imaging features, COVID-19 and other viral pneumonias, bacterial pneumonia and some lesions share some common imaging features. In some cases, it is difficult to differentiate COVID-19 from them by imaging alone and first clinical manifestations, contact history and laboratory tests should also be considered to make the final diagnosis [12-15]. In contrast to the PCR of SARS-CoV-2, which is restricted by false-negative results and detection limitations, chest CT, which is simple to perform and readily available, can quickly detect lung lesions and make imaging diagnosis at the early stage. Therefore, it has great value in early screening, differential diagnosis and disease severity assessment of COVID-19 [12-15]. In addition to previous results, we must highlight the importance of using a broad spectrum molecular diagnostic panel for rapid detection of the most common respiratory pathogens, in order to improve evaluation and clinical management of patients with respiratory syndrome consistent with COVID-19. This is important, where alternative diagnosis may clarify an individual patient’s risk and may allow adjusting public health containment measures. Nevertheless, it is mandatory to maintain high level of attention with respect to this new emergent pathogen and health authorities should remain vigilant, increasing their capacity for surveillance and constantly reviewing their pandemic preparedness plans [5].

## Conclusion

Differential diagnosis is very important to early quarantine suspected, for the reason that delayed care may affect the prognosis, proper care and the evolution of these patients with a higher risk of severe complications, decompensations and even death. With exposure history and common respiratory symptoms in this epidemic period shared with other respiratory diseases and infections, we emphasize the importance of good screening protocols, rapid detection of SARS-CoV-2 and other most common respiratory pathogens; and performing chest CT in the early stages, which may help for a better control of COVID-19 spread and avoid delayed care of other lung diseases.

### What is known about this topic

- Coronavirus disease 2019 (COVID-19) is caused by severe acute respiratory syndrome coronavirus 2 (SARS-CoV-2);
- Its common symptoms are nonspecific and shared by many other frequent infectious diseases of the respiratory tract and other respiratory tract diseases.

### What this study adds

- The importance of good screening protocols, rapid detection of SARS-CoV-2 and other most common respiratory pathogens; and performing chest CT in the early stages;
- Differential diagnosis of COVID-19 is very important to early quarantine suspected patients, which may help to avoid delayed care of other lung diseases; while maintaining vigilance towards this pandemic spread.
